# Optimizing Gluten Extraction Using Eco-friendly Imidazolium-Based Ionic Liquids: Exploring the Impact of Cation Side Chains and Anions

**DOI:** 10.1021/acsomega.3c08683

**Published:** 2024-04-02

**Authors:** Wen-Hao Chen, Chuan-Chih Hsu, Hui-Yin Huang, Jong-Yuh Cherng, Yu-Cheng Hsiao

**Affiliations:** † Research and Development Group, Yen Hao Holding Company, Tainan 11031, Taiwan; ‡ Division of Cardiovascular Surgery, Department of Surgery, School of Medicine, College of Medicine, Taipei Medical University, 250 Wuxing Street, Taipei 11031, Taiwan; § Division of Cardiovascular Surgery, Department of Surgery, Taipei Medical University Hospital, 250 Wuxing Street, Tai-pei 11031, Taiwan; ∥ Graduate Institute of Biomedical Optomechatronics, College of Biomedical Engineering, Taipei Medical University, Taipei 11031, Taiwan; ⊥ Department of Chemistry and Biochemistry, National Chung Cheng University, Chia-yi 62102, Taiwan; # Stanford Byers Center for Biodesign, Stanford, California 94305, United States; ¶ Cell Physiology and Molecular Image Research Center, Wan Fang Hospital, Taipei Medical University, Taipei 11031, Taiwan

## Abstract

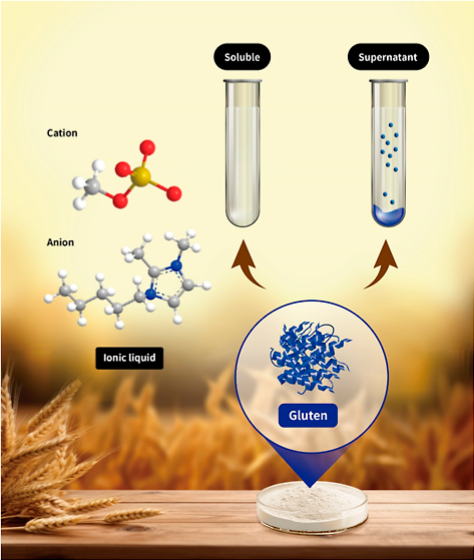

Gluten is a well-known food allergen globally, and it can induce immune responses in celiac- and nonceliac gluten-sensitive patients. The gliadin proteins from gluten have a special amino acid sequence that make it hydrophobic. One way to deal with gluten allergies is to provide a gluten-free diet. The hydrophobic characteristic of gliadin makes gliadin detection more difficult. An analyst needs to use an organic solvent or multiple processes to denature gluten for extraction. Although organic solvents can rapidly extract gluten in a sample, organic solvent also denatures the antibody and induces false biotest results without buffer dilute, and the accuracy will reduce with buffer dilute. An ionic liquid (IL) is a highly modifiable green chemical organic salt. The imidazolium has a cationic structure and is modified with different lengths (*C* = 0, 1, 3, 5, 7, 9, and 12) of carbon side chains with organic and inorganic anions [methanesulfonate (MSO), Cl^–^, F^–^, NO_3_^–^, HSO_4_^–^, and H_2_PO_4_^–^] to make different kinds of ILs for testing the solubility of gliadin. Different IL/water ratios were used to test the solubility of gluten. We measured the solubility of gliadin in different imidazolium ILs, and the kinetic curve of gliadin dissolved in 1% [C5DMIM][MSO]_aq_ was conducted. We also used circular dichroism spectroscopy and an enzyme-linked immunosorbent assay to measure the gliadin structure and the effect of binding with an antibody after 1% [C5DMIM][MSO]_aq_ treatment. An 2,3-bis-(2-methoxy-4- nitro-5-sulfophenyl)-2*H*-tetrazolium-5-carboxanilide (XTT) assay was used to test the toxicity of [C5DMIM][MSO]_aq_ in N2a cells. In our research, 1% [C5DMIM][MSO]_aq_ produced a good solubility of gluten, and it could dissolve more than 3000 ppm of gluten in 5 min. [C5DMIM][MSO]_aq_ did not break down the gluten structure and did not restrict antibody binding to gluten, and more importantly, [C5DMIM][MSO] did not exhibit cell toxicity. In this report, we showed that [C5DMIM][MSO] could be a good extraction solution applied for gluten detection.

## Introduction

Around 0.5∼6% of people worldwide have celiac disease or nonceliac gluten sensitivity (NCGS).^1−5^ Celiac disease and NCGS are food allergy diseases, and the reason comes from gluten-induced immune responses that attack the body.^6,7^ Gluten allergy symptoms include bloating, chronic diarrhea, depression, anxiety, etc. It raises the risks of enteropathy-associated T-cell lymphoma, non-Hodgkin’s lymphoma, and adenocarcinoma of the small intestines when celiac patients do not control their gluten intake.^8−10^

Gluten is a major food allergen worldwide, and it comprises two major proteins: glutenin and gliadin.^11^ Glutenin and gliadin are both insoluble in water. Some research claimed that gliadin appears to be the primary cause of celiac disease. Gluten contains gliadin groups (alpha/beta, omega, and gamma) and glutenin subunits (high-molecular weight and low-molecular weight). Regardless of the kind of gliadin, gliadins are capable of aggregating into larger oligomers and interacting with other gluten proteins due to large hydrophobic sections,^12,13^ poly-Q, and repetitive sequences.^14−16^ These sections are likely to aggregate hydrophobically,^17^ separate in the liquid–liquid phase, potentially form β-sheet aggregates,^18^ or simply become entangled by their structural properties. Detection of gluten is difficult due to the effects of hydrophobicity and aggregation. Analysts need to use organic solvents or spend more time pretreating a sample to extract gliadin from a sample. Recently, scientists have tried to use certain enzymes to hydrolyze gluten to decrease its toxicity,^19−21^ but still no useful process has been found to eliminate the toxicity^22,23^ according to Food and Drug Administration publication in 2022.

Although food technology keeps progressing, the only good way to treat gluten allergies so far is to avoid consuming gluten. Generally, for gluten analysis, an enzyme-linked immunosorbent assay (ELISA) is the main gluten detection system. An ELISA has high specificity for gliadin antibodies. However, gliadin is difficult to dissolve in water solutions, especially in phosphate-buffered saline (PBS buffer).^24^ Analysts need to pretreat samples with 75% alcohol or spend time heating the sample in solutions.^25−27^ Alcohol is detrimental to antibodies as it can induce antibody mutations, causing them to lose their function. Therefore, the analyst needs to transfer alcohol to a buffer system (e.g., PBS buffer) to reduce its effects on the antibody, but the accuracy will reduce with buffer dilute. It is a complicated process that takes time to pretreat samples, regardless of the process chosen.

Ionic liquids (ILs) are synthesized by organic cations and organic/inorganic anions.^28,29^ IL function can be varied by modifying side chains, and they can have different physical and chemical effects by changing the anions.^29^ ILs can be applied to heavy metals,^30^ little molecular^31^ extraction, and also to stable protein structures.^32^ Based on the ability to modify ILs, we designed and synthesized different lengths of side chains and anions for the rapid extraction of gluten from samples. In this report, we used imidazolium as the major structure, modified by adding different lengths of carbon side chains for interactions with hydrophobic sides of gluten, and changed the anion to increase the solubility of gluten.

ILs synthesized provide the interaction with gliadin protein. Ionic forces come for the salt of the hydration radius to interact with a protein. van der Waals forces arise for interactions with carbon lengths. Generally, the van der Waals force increases with length. Since gliadin has special amino acid sequences like poly glutamine and continuous poly proline, these special amino acid sequences induce protein–water insolubility and cause the fiber structure to easily aggregate.^33,34^ For this special amino acid sequence, we synthesize a suitable side chain of ILs to bind to the gliadin protein, to make gliadin soluble in water for a safe and effective analysis to gliadin detection.

## Results and Discussion

### Solubility of Gliadin in [C5DMIM][MSO]_aq_

Ionic solutions of [pentyl dimethyl imidazolium][methyl sulfonic] ([C5DMIM][MSO]) at concentrations of 0.05, 0.1, 1, 2, 5, and 10 wt % in water were prepared in sample vials. Here, We choose gluten from wheat to be a sample to simulate the gliadin in food and used the gliadin ELISA kit to measure the concentration of gliadin.

**Figure 1:**
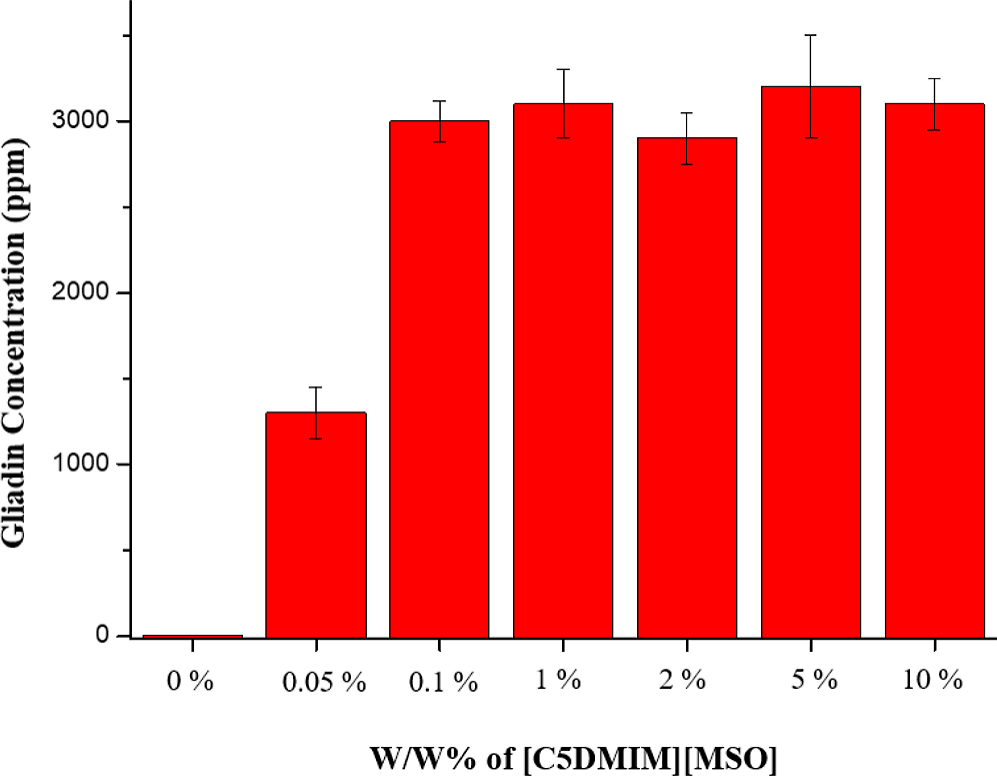
Gliadin solubility in the [C5DMIM][MSO] water solution.

3 g of gluten (an excess amount, gluten from wheat purchased from Sigma-Aldrich) was added to 10 mL of each [C5DMIM][MSO] ionic solution, mixed homogeneously by stirring for 30 min, and allowed to sit for 5 min for the test example. All sample solutions were centrifuged at 8500 rpm for 3 min and filtered through a 0.22 μm pore size filter. Water (0 wt %) was used as the control group. After that, a Wheat/Gluten (Gliadin) ELISA kit (Crystal Chem, AOAC no. 011804) was used to determine the concentration and solubility of gliadin. Since the limit of the Wheat/Gluten (Gliadin) ELISA kit was about 100 ppm, the gliadin concentration was determined by diluting sample solutions by at least 50-fold with a corresponding ionic solution in order to calculate their gliadin concentrations. As shown in Figure 1, gliadin solubility increased when the percentage of [C5DMIM][MSO] was raised from 0 to 0.1%, and the maximum value of gliadin solubility was at 3000 ppm of 1% [C5DMIM][MSO]_aq_. There was no significant difference in gliadin solubility when [C5DMIM][MSO]_aq_ was raised to 10%.

### Side-Chain Effects on Gliadin Solubility in 1 wt % of an IL Water Solution

The special function originates from modifications of the IL side chains. In this manuscript, we synthesized different side-chain lengths to test the IL solubility of gluten. In recent years, some reports showed that longer side chains and anions of ILs could penetrate cell membranes and damage cells and macrobiotics.^35,36^ For this reason, we tried to synthesize more environmentally friendly ILs and applied them to gluten extraction.

We synthesized and prepared 1% IL water solutions of {[hydrogen dimethyl imidazolium][methyl sulfonic] ([HDMIM][MSO]), [trimethyl imidazolium][methyl sulfonic] ([TMIM][MSO]), [propyl dimethyl imidazolium][methyl sulfonic] ([C3DMIM][MSO]), [C5DMIM][MSO],[heptyl dimethyl imidazolium][methyl sulfonic] ([C7DMIM][MSO]), [nonyl dimethyl imidazolium][methyl sulfonic] ([C9DMIM[MSO]], and [dodecyl dimethyl imidazolium][methyl sulfonic] ([C12DMIM][MSO])) in sample vials. Using the same process, the gliadin solubility in different IL solutions was measured.

**Figure 2:**
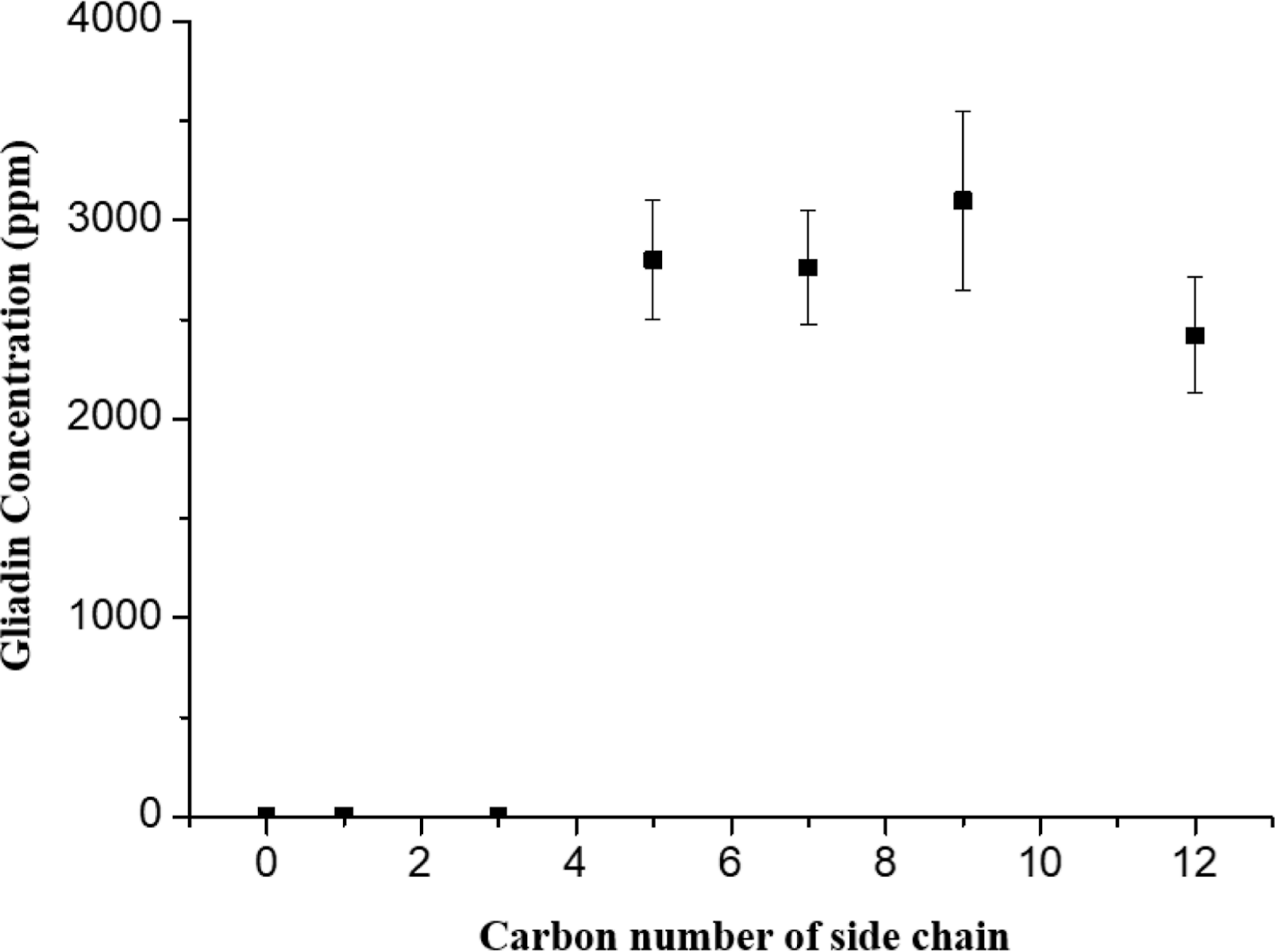
Side-chain effect of imidazolium with methanesulfonic acid anions on the gliadin solubility test.

As shown in Figure 2, gliadin had a higher solubility in the [C5DMPIM][MSO], [C7DMIM][MSO], [C9DMIM][MSO], and [C12DMIM][MSO] IL solutions, in which the corresponding groups attached to the 3-position of 1,2-dimethyl imidazolium had different carbon numbers of 5, 7, 9, and 12, respectively. The [C9DMIM][MSO] and [C12DMIM][MSO] ILs had lower solubilities but higher viscosities, which might be disadvantageous. However, decreasing the length of the carbon chains also reduced toxicity toward the environment.^35,36^

### Solubility of Gliadin in 1 wt % Ionic Solutions of Ionic Compounds with Different Anions

Different anions will have different effects in ILs. The hydrophobicity, hydrophilicity, and melting and boiling points can be controlled by different anions of ILs.^29,37^ In this report, we changed the anions to test the gliadin solubility of the ILs. We synthesized and prepared 1% IL water solutions of [C5DMIM][Cl], [C5DMIM][F], [C5DMIM][MSO], [C5DMIM][HSO_4_], [C5DMIM][NO_3_], and [C5DMIM][H_2_PO_4_] in sample vials. Using the same process and ELISA, the gliadin solubility in different IL solutions was measured.

**Figure 3:**
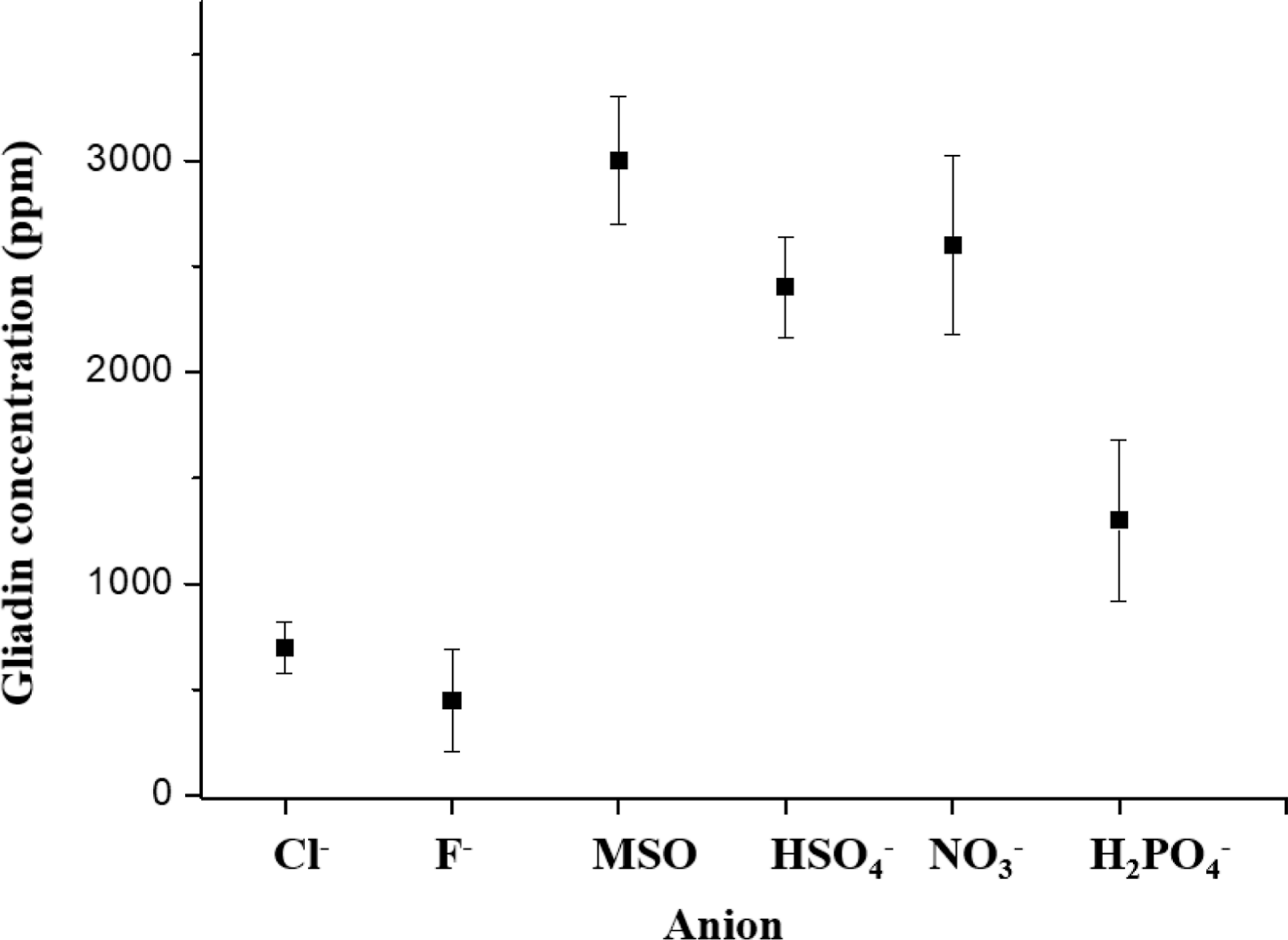
Anion effects on the gliadin solubility test with [C5DMIM].

As shown in Figure 3, there is bad solubility of gliadin in [C5DMIM][Cl]_aq_ and [C5DMIM][F]_aq_. [C5DMIM][MSO]_aq_ shows signification of great solubility of gliadin in this report. Gliadin solubility still had a good effect on certain inorganic anions (HSO_4_^–^, NO_3_^–^, and H_2_PO_4_^–^).

### Kinetics of the Solubility of Gliadin in 1 wt % [C5DMIM][MSO]_aq_

ILs hold great potential as a solvent for organic transformations.^28^ In this report, we test the solubility of gliadin in 1 wt % [C5DMIM][MSO]_aq_ and PBS buffer in different time points.

**Figure 4:**
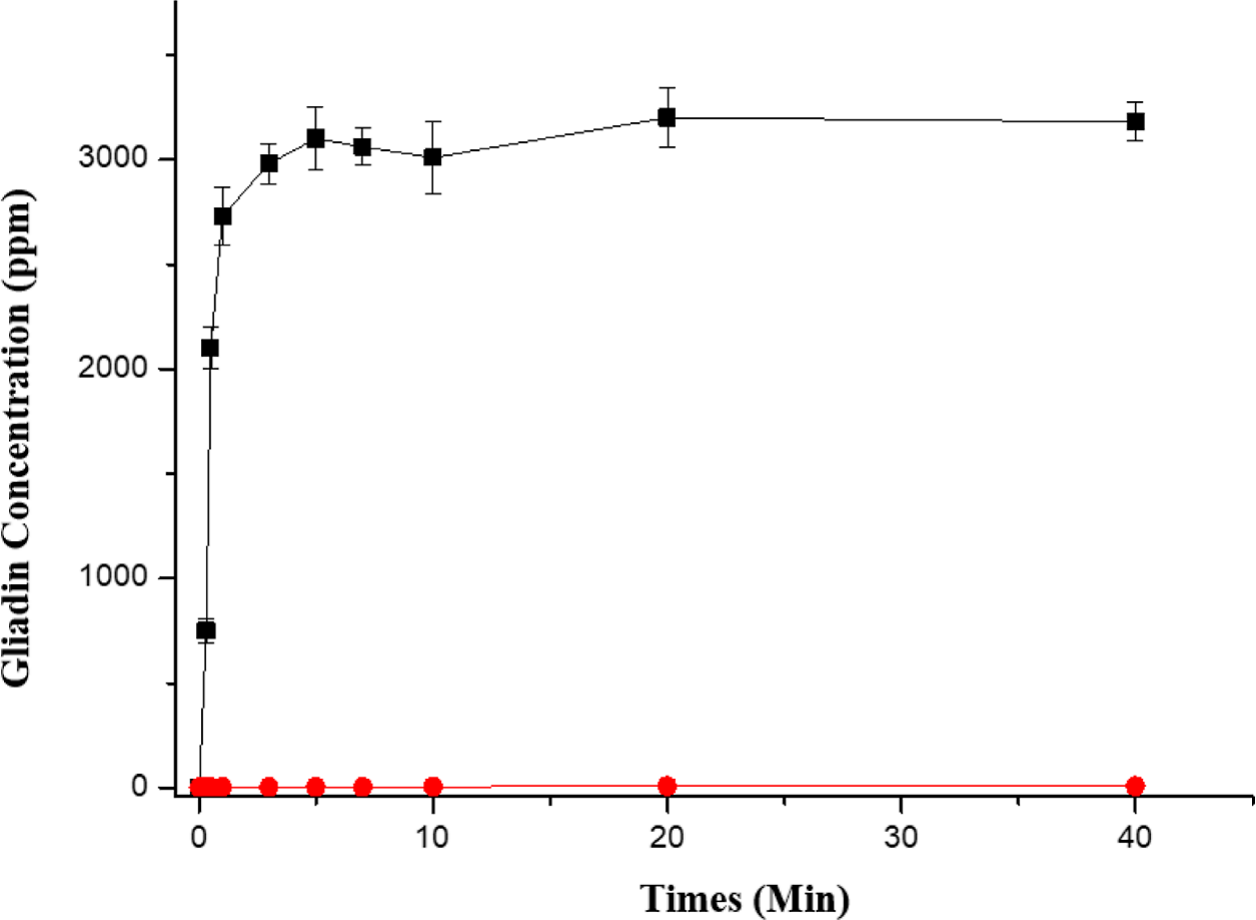
Kinetic curve of gliadin dissolved in 1% [C5DMIM][MSO]aq (black squares) and PBS buffer (red dots).

As shown in Figure 4, the solubility of gliadin with PBS buffer extraction was less than 5 ppm with a longer time for extraction under room temperature (red dot), the solubility of gliadin was more than 700 ppm in 30 s after [C5DMIM][MSO]_aq_ extraction, and the solubility of gliadin reached its maximum in 5 min (black square) under room temperature. These data showing the ILs of [C5DMIM][MSO]_aq_ have a high effect on solubility of the gliadin.

### Structure of Gliadin with/Without IL Extraction

Circular dichroism (CD) spectroscopy is very sensitive to the secondary structures of polypeptides and proteins. It is usually used to study the secondary structure (α helix/ta sheet) of peptides or proteins. In this manuscript, we used CD spectra to measure the secondary structure of gliadin with/without [C5DMIM][MSO] extraction. A gliadin sample without an IL solution (20 μM) was prepared from a standard gliadin solution (1 mg/mL) in PBS buffer, which was directly prepared using commercially available gliadin (Leadgene). For the gliadin sample without an IL solution (20 μM), 1 mL of the solvent was removed by a vacuum, and then, 1 mL of 1% of the IL ([C5DMIM][MSO]) solution was added to prepare the sample with IL extraction.

**Figure 5:**
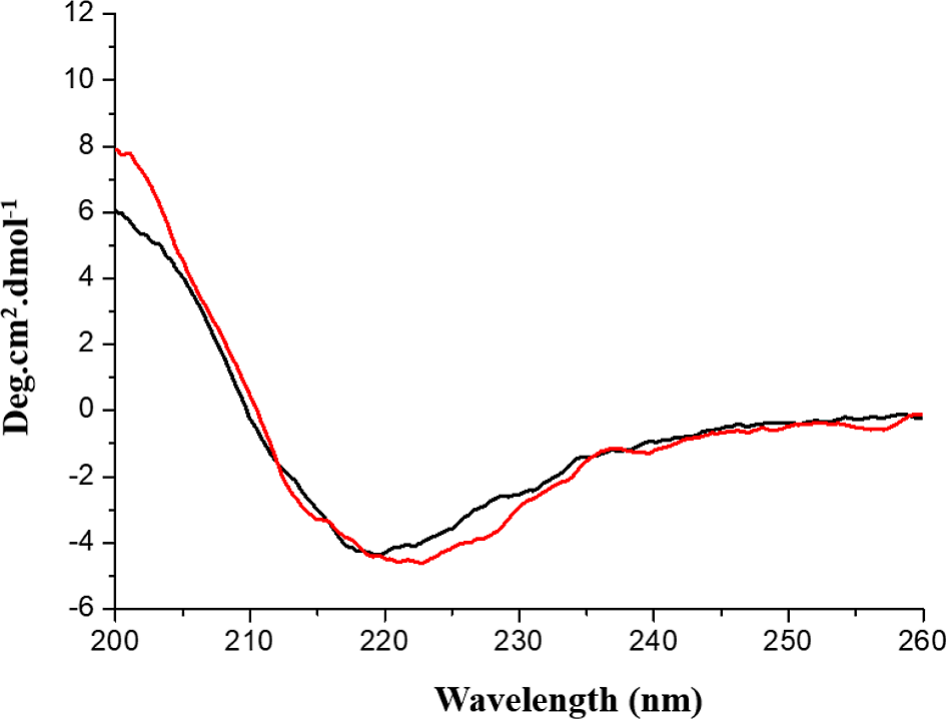
CD spectra of gliadin with (black line) and without (red line) [C5DMIM][MSO] treatment.

A CD spectrometer (Jasco J-815) was used for the structural analysis of the abovementioned sample solutions obtained by gliadin-rice noodle sample extraction and a commercially available gliadin product. As shown in Figure 5, a β sheet structure was evident between the extracted gliadin and commercially available gliadin (not extracted with the currently conceived ionic solution). Figure 5 shows that gliadin with wt1% [C5DMIM][MSO] (red line) was less than gliadin with 1% [C5DMIM][MSO] (black line) at 220–230 nm, which indicates that the IL interacted with gliadin and changed a little bit of its structure. However, generally, the CD spectra were similar between gliadin with and that without [C5DMIM][MSO] treatment, which means that the secondary structure of gliadin did not significantly differ after 1% [C5DMIM][MSO] treatment of gliadin.

### Biochemical Analysis of Gliadin Resolved in 1 wt % Ionic Solutions of 1 wt % [C5DMIM][MSO]

**Figure 6:**
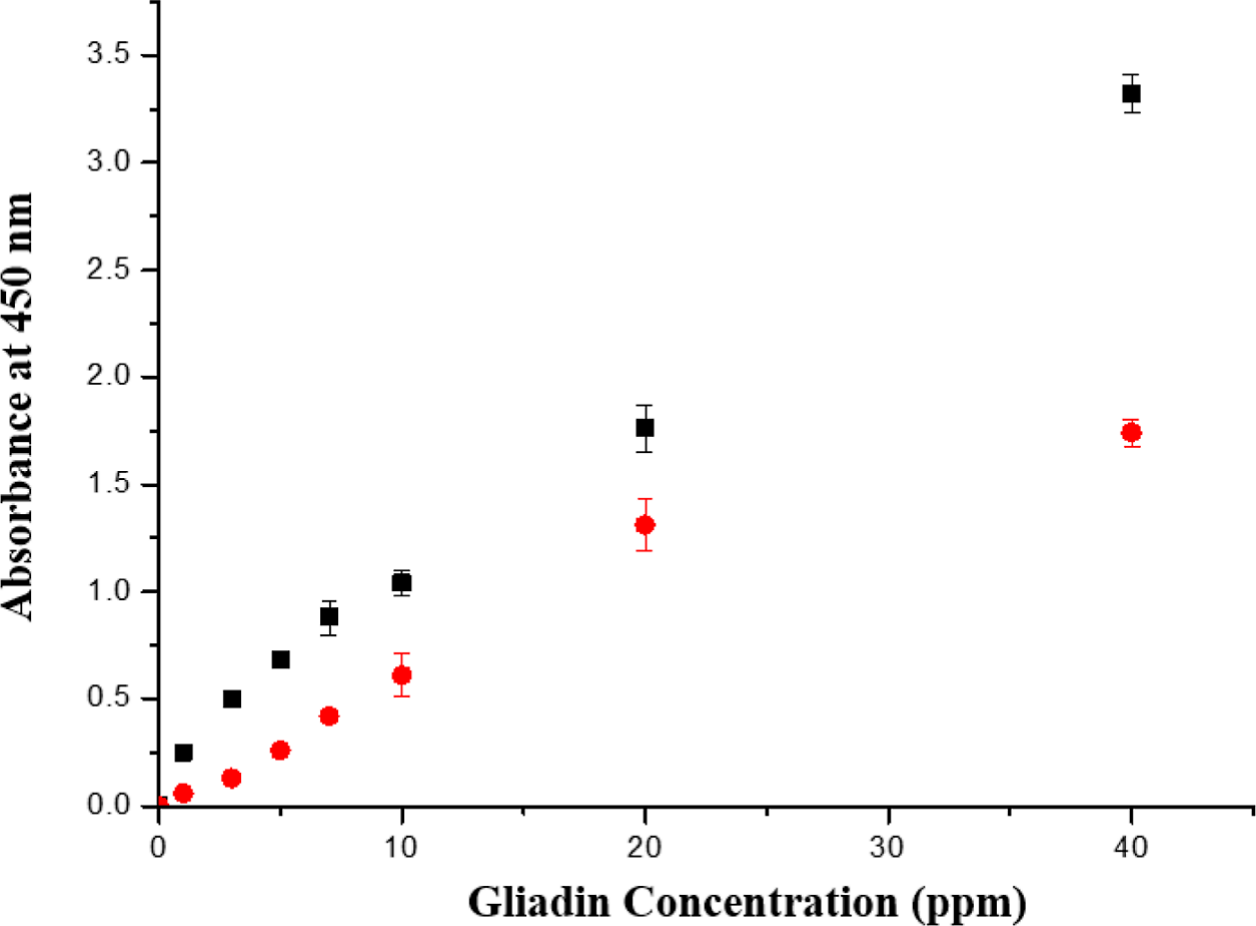
ELISA assay of gluten with 1% [C5DMIM][MSO] (black squares) and a 1% SDS solution (red dots).

Biosensors are a very important technology globally, and this technology is widely used for disease diagnosis, detection of microorganisms and viruses, and all kinds of biomarker tests. The ELISA is widely used in biosensor issues because it is specific and sensitive for target proteins according to the antibodies used. Certainly, ELISA is the major biosensor for gliadin now. Data are shown in Figure 6.

In this report, we coated the antibody on an ELISA plate and purchased antibody conjugated with horseradish peroxidase (HRP) for biochemical assay. We prepare different concentrations of gliadin solution with IL/sodium dodecyl sulfate (SDS) and test the gliadin solution by ELISA. In the test, different concentrations of gliadin solution show different intensities in biochemical assay.

As shown in Figure 5, the regression line had an *R*^2^ value of 0.9942. This indicates that [C5DMIN][MSO]aq interacted with gliadin to raise the solubility, and the antibody could still bind with gliadin after [C5DMIM][MSO] interaction.

For the control test (1% SDS for extraction), the absorbance was lower than the [C5DMIM][MSO] model, and its curve had a maximum at 20 ppm, which probably came from the strong cleaning effect of SDS to remove the original antibody coated onto the plate.

### Gliadin Recovery Rate Study with [C5DMIM][MSO]

The recovery rate is an important factor in extraction systems, and it will show the effect of extracting the target in a sample. In this report, we tested the recovery of gliadin by 1% [C5DMIM][MSO]_aq_.

Rice noodles are a well-known food without gluten, and it was selected to be a sample for the recovery test. Rice noodles were measured without gliadin by ELISA at first. 200 ppm of gliadin solution was added on rice noodles, and the solution was removed by vacuum to prepare the standard of gliadin sample.

1% of [C5DMIM][MSO]_aq_ for extraction of the gliadin from standard gliadin sample was used, and the gliadin concentration was measured by ELISA. The data are shown in Table 1.

**Table 1:** Percent Recovery = [Extraction of Gluten by the IL Solution/Standard Gluten Concentration] × 100%

	standard gluten (ppm)	extraction gliadin (ppm)	recovery (%)^1^
1	200	196	98
2	200	196	98
3	200	197	98
4	200	194	97
5	200	192	96
average	200	195	97.5

The recovery rate 100% was defined as 200 ppm of gliadin in the five times of the recovery test by 1% [C5DMIM][MSO]_aq_. The average recovery rate of the 1 wt % ionic solution of [C5DMIM][MSO]_aq_ was 97.5%.

### Biocompatibility of IL Solutions of [C5DMIM][MSO]_aq_

In recent years, the IL structure was reported to have cell toxicity toward cells and bacteria due to its long side-chain effect (ref). For this reason, we tested the cell toxicity of [C5DMIM][MSO] in this report. 2,3-Bis-(2-methoxy-4- nitro-5-sulfophenyl)-2*H*-tetrazolium-5-carboxanilide (XTT) is commonly used to test nonradioactive quantification of cellular proliferation, viability, and cytotoxicity. The sample material was either adherent or suspended cells cultured in 96-well microplates. An increase in the number of living cells resulted in an increase in the overall activity of mitochondrial dehydrogenase in the sample. This increase was directly correlated to the amount of orange formazan formed, as monitored by the absorbance.

In this report, we chose mouse N2a neuroblastoma cells to test the toxicity of [C5DMIM][MSO].

**Figure 7:**
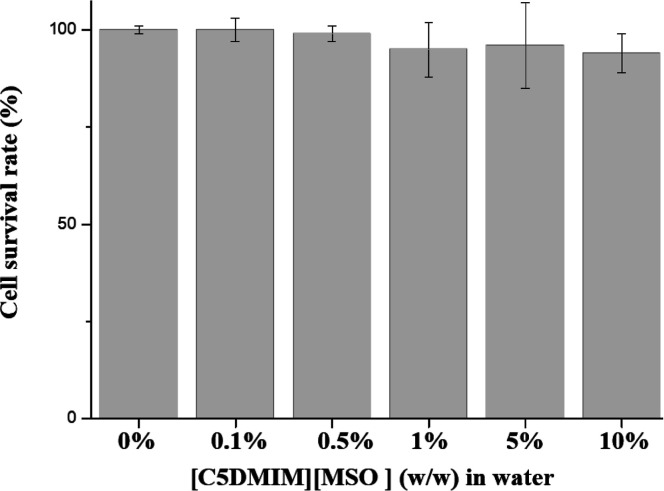
Cell toxicity with different concentrations of [C5DMIM][MSO]_aq_.

As shown in Figure 7, survival rates of cells in the [C5DMIM][MSO] ionic solutions at all concentrations were higher than 97%. This indicates that the ionic compounds in the present formulation had good biocompatibility.

## Conclusions

Wheat is a major food for humans globally, but around 1∼6% of people in the world have wheat allergy issues. Gluten from wheat can induce an inflammatory immune response in those patients. There are two major proteins, glutenin and gliadin, in gluten. In recent years, some research claimed that gliadin is the major protein that induces the inflammatory immune response in patents. In food tests, gluten detection requires a lot of time for pretreating samples because gliadin is water insoluble. For this reason, we tried to synthesize an IL and applied it to rapid gluten extraction. The comparison of several methods of the gliadin extraction process is shown in Supporting Information Table S2.

In this report, we synthesized ILs with different lengths of side chains and different anions of the imidazolium base ILs to study the side chains and anion effects of imidazolium ILs applied to dissolve gliadin, and we purchased a gluten/gliadin ELISA kit to measure the extraction effect of imidazolium-based IL water solutions.

As to the side-chain effect of the gliadin solubility test, the ILs of [HDMIM], [TMIM], [C3DMIM], [C5DMIM], [C7DMIM], [C9DMIM], and [C12DMIM] cations with MSO anions were synthesized to study gliadin solubility with an imidazolium base of a 1% IL water solution, and we found that the 1% IL water solution produced good gliadin solubility when the carbon chain was greater than 5 on imidazolium. In the anion effect test, we synthesized [C5DMIM] with different anions (F, Cl, NO_3_, HSO_4_, H_2_PO_4_, and MSO), and [C5DMIM] with MSO anions produced the best gliadin solubility. In the kinetic curve of gliadin solubility, [C5DMIM][MSO] could dissolve more than 2500 ppm of gliadin in 1 min, and over 3000 ppm of gliadin could be dissolved in the IL water solution in 3 min.

In this report, we measured the secondary structure of gliadin with and without [C5DMIM][MSO] by CD spectroscopy and found that the spectra did not significantly differ with or without [C5DMIM][MSO]. We measured the antibody–gliadin interaction by ELISA after 1% [C5DMIM][MSO] extraction, and the results showed good linearity of different concentrations of gliadin with [C5DMIM][MSO]. It showed that [C5DMIM][MSO] was not restricted by binding of antibodies with gliadin. In the recovery test, the average recovery rate was 97.5% from rice noodles with gliadin added with 1% [C5DMIM][MSO].

Generally, the toxicity of cells will increase when the number of side chains increases. For this reason, we selected [C5DMIM][MSO] to test the cell toxicity, but we found no significant cell toxicity from [C5DMIM][MSO] in the N2a model. Those data showed that [C5DMIM][MSO] can be an extraction buffer for extracting gliadin from food. This technology can increase the effect of detecting gliadin and reduce the number of organic solvents and multiple processes required to pretreat samples. It could be a great development to protect celiac patients.

## Materials and Methods

### Reagents and Solvents

All solutions and samples were prepared by using deionized water with a resistivity of 18.2 Ω cm^–1^ from a Millipore Milli-Q water purification system (Millipore). Gluten from wheat was purchase from Sigma-Aldrich. 1,2-Dimethyl imidazole (98%), 1-fuloro pentane, methanol (99%), propanol (99%), pentanol (99%), heptanol (98%), nonanol (98%), and dodecanol (98%) were purchased from ACOS; dichloromethane (99%) was purchased from Tedia (98% purity); acetonitrile (99%), ether (99%), fetal bovine serum (FBS), gluten from wheat, methanesulfonic acid (99), methane sulfonic chloride (99%), thionyl chloride (98%), silver nitride (95%), silver dihydrogen phosphate (99%), and silver bisulfate (99%) were purchased from Sigma-Aldrich; antigliadin mouse IgG 2F (1 mg/mL) was purchased from Antaimmu; antigliadin human IgA 3B7 (1 mg/mL) and a gliadin solution (1 mg/mL) were purchased from Leadgene; HRP was present in the MagicLink HRP antibody conjugation kit; 3,3′,5,5′-tetramethylbenzidine (TMB) and hydrogen peroxide (H_2_O_2_) reagent were purchased from TCI; and the Wheat/Gluten (Gliadin) ELISA kit was purchased from Crystal Chem (AOAC no. 011804). DEMD and XTT reagents were purchased from Thermo Fisher.

IL synthesis is given in the Supporting Information, and the product yield is given in Table S1.

### Antibody Conjugated with HRP

We prepared 1 mg/mL purified antibody in PBS buffer. The antibody solution was directly added to the vial of Magic NHS (component A) and mixed well by repeatedly pipetting a few times or vortexing the vial for a few seconds. The antibody-labeling reaction mixture was kept at room temperature for 60 min. The sample was placed in a filter device, and a microcentrifuge (14,000*g* for 3 min) was used to purify the antibody (the liquid from the filter was discarded), which was repeated twice. Then, the labeled antibody was collected from the filter device into a microcentrifuge tube.

The LINK-HRP solution was made by adding 250 μL of deionized water into the vial of LINK-HRP (component B) and mixed well by repeatedly pipetting a few times or vortexing the vial for a few seconds.

The antibody conjugate was stored at >0.5 mg/mL in the presence of a carrier antibody [e.g., 0.1% bovine serum albumin (BSA)]. For longer storage, the HRP-antibody conjugates could be lyophilized and stored at ≤ – 20 °C.

### CD Measurements of Gliadin

Gliadin was present in PBS buffer (70 mm KCl and 20 mm Na_3_PO_4_, at pH 7.26) at 37 °C. The secondary structure of the resulting gluten was recorded in a 1 mm quartz cuvette using a J-815 CD spectrometer (Jasco, Japan).

### Cytotoxicity Assay

N2A cells were seeded into a 96-well plate and treated with different concentrations of the IL solution for 2 h, and then, the solution was removed, and cells were reincubated for 24 h. The XTT reagent was added, and cells were incubated at 37 °C for 2 h. The absorbance was measured with a TECAN Infinite 200 PRO.

### Biochemical Assay

In this report, we used an ELISA to test gliadin binding with an antibody after 1% [C5DMIM][MSO] and control (1% SDS) treatment. The 1% IL solution of [C5DMIM][MSO] in water was first prepared. Specific amounts of gliadin were added to the 1 wt % IL solution of [C5DMIM][MSO] to prepare gliadin sample solutions at 1, 2, 3, 5, 7.5, 10, 20, and 40 ppm of gliadin. After that, gliadin concentrations were confirmed by using a Wheat/Gluten (Gliadin) ELISA kit.

A 96-well empty ELISA plate was coated with 1 μg/mL antigliadin mouse immunoglobulin G (IgG; 2F, purchased from Antaimmu) and then blocked with 1% BSA to obtain an ELISA plate for gliadin examination. Gliadin sample solutions were, respectively, added to wells of the ELISA plate to react for 5 min, and then, the wells were washed by PBS buffer. After removing excess solution, 0.1 μg/mL antigliadin human IgA-HRP (antigliadin human IgA 3B7 purchased from Leadgene and HRP added by the MagicLink HRP antibody conjugation kit) was added to react for 15 min, the plate was washed, TMB/H_2_O_2_ reagent (T3854, purchased from TCI) was added to react for 10 min, and a 0.5 mol/L sulfuric acid aqueous solution was added to terminate the reaction. The absorbance at 450 nm of all wells on the plate was examined by an ELISA reader (TECAN Infinite 200 PRO).

### Recovery Test

Rice noodles are a well-known food without gluten. In this report, we chose rice noodles as a substrate to test the recovery of gliadin by 1%[C5DMIM][MSO]. A 1 wt % IL solution of [C5DMIM][MSO] in water was first prepared. Hydrophobic proteins were traditionally extracted by an alcohol solution, so a 75 wt % ethanol solution was also prepared for this test.

3 g of bread flour (Blue Jacket Strong Flour, Lien Hwa Milling) was mixed with 10 mL of 75 wt % ethanol for extraction at room temperature for 5 min to produce standard sample solutions. The sample solutions were centrifuged at 8500 rpm for 3 min and filtered with a 0.22 μm pore size filter to obtain filtered samples. After filtration, the Wheat/Gluten (Gliadin) ELISA kit (Crystal Chem, AOAC no. 011804) was used to determine the gliadin concentrations in the filtered samples. The gliadin concentration was determined by diluting the sample solutions by at least 50-fold with the corresponding ionic solution in order to calculate their gliadin concentrations. In both groups of 75 wt % ethanol extraction, gliadin was higher than 2000 ppm.

In addition, 40 g of dried gluten-free rice noodles (Organic Rice Noodles, Yuan Shun Food) was first soaked in water to rehydrate the noodles. After rehydration, the rice noodles were drained and soaked in 10 mL of a 200 ppm solution of gliadin in 100% ethanol in a container at room temperature to produce gliadin-rice noodles. The gluten-free rice noodles had a very large specific surface area (total surface area per unit of bulk volume), and most gliadin was adsorbed by the rice noodles.

After the ethanol solvent was evaporated, the gliadin-rice noodles were lyophilized in a container to give a gliadin-rice noodle sample. 10 mL of a 1 wt % IL solution of [C5DMIM][MSO] (equal volume with the 200 ppm of gliadin solution in ethanol) was added to the lyophilized gliadin-rice noodles for extraction at room temperature for 5 min to produce test sample solutions. The sample solutions were centrifuged at 8500 rpm for 3 min and filtered with a 0.22 μm pore size filter to obtain filtered samples. All groups were repeated five times. The concentration of gliadin was also determined with the Wheat/Gluten (Gliadin) ELISA kit (Crystal Chem, AOAC no. 011804), wherein at least five-fold diluted sample solutions with a 1 wt % IL solution of [C5DMIM][MSO] were used.

### Biocompatibility of [C5DMIM][MSO]_aq_

N2a cells were cultured and maintained in high-glucose Dulbecco’s modified Eagle’s medium (DMEM) with l-glutamine and sodium pyruvate (DMEM-HPA-P10, Capricorn Scientific) containing 10% FBS. N2a cells were seeded in six-well plates at a density of 2 × 10^5^ cells/well overnight. Then, the medium was replaced with test high-glucose DMEM medium containing 0.1, 1, 2, 5, or 10 wt % [C5DMIM][MSO], and cells were incubated in an incubator (37 °C, with a 5% CO_2_ humidified atmosphere) for 6 h. The test medium was removed, the cells were washed, and fresh high-glucose DMEM containing 10% FBS was added and incubated for another 12 h. After that, cells were subjected to an XTT test (X12223, purchased from Thermo Fisher) to determine the cell survival rate.

### Kinetic of Solubility of Gliadin in 1% [C5DMIM][MSO]_aq_ and PBS Buffer

We prepared 1% [C5DMIM][MSO]_aq_ and PBS buffer in sample vials, separately added 3 g of gluten into the vials, homogeneously mixed it by a vortex mixer shaking the vials around 10 min, and allowed it to sit for various intervals (0.5, 1, 2, 3, 5, 10, 20, and 30 min). We used a 0.22 μm filter to remove precipitates, and the gliadin solubility was measured with a gliadin ELISA kit. As the control test, a PBS buffer was selected to substitute for [C5DMIM][MSO]_aq_. As the control test, kinetics of gliadin dissolution in PBS buffer was measured. We used the ELISA to measure the gliadin concentration after pretreatment.

## Supporting Information Available

Yield and product state for imidazolium IL with MSO anion and comparison of extraction methods (PDF)
