# Supplementary material for: Optimizing Gluten Extraction Using Eco-friendly Imidazolium-Based Ionic Liquids: Exploring the Impact of Cation Side Chains and Anions
Source: ACS Omega. 2024 Apr 2;9(15):17028–35. doi: 10.1021/acsomega.3c08683 (PMC11025095; doi:10.1021/acsomega.3c08683)
Supplement: Supplementary file 1 — ao3c08683_si_001.pdf [file ao3c08683_si_001.pdf]

Optimizing Gluten Extraction Using Eco-Friendly Imidazolium-Based Ionic Liquids: Exploring the Impact of Cation Side Chains and Anions

Wen-Hao Chen[ad+], Chuan-Chih Hsu[bc+], Hui-Yin Huang[a], Jong-Yuh Cherng[e]*, Yu-Cheng Hsiao[adfg]*

a.Research and Development Group, Yen Hao Holding Company, Tainan, Taiwan.
b Division of Cardiovascular Surgery, Department of Surgery, School of Medicine, College of Medicine, Taipei Medical University, 250 Wuxing St., Taipei 11031, Taiwan
c Division of Cardiovascular Surgery, Department of Surgery, Taipei Medical University Hospital, 250 Wuxing St., Tai-pei 11031, Taiwan
d Graduate Institute of Biomedical Optomechatronics, College of Biomedical Engineering, Taipei Medical University, Taipei 11031, Taiwan.
e Department of Chemistry and Biochemistry, National Chung Cheng University, Chia-yi, Taiwan.
f Stanford Byers Center for Biodesign, Stanford, CA, USA
g Cell Physiology and Molecular Image Research Center, Wan Fang Hospital, Taipei Medical University, Taipei 11031, Taiwan

S1. Organic synthesis and NMR data.

Ionic liquid synthesis:[1,2]

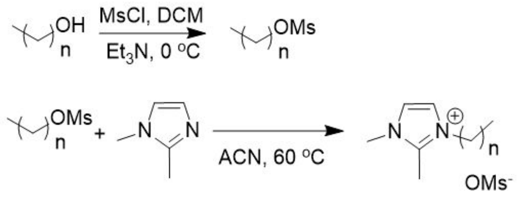

Scheme S1. Synthesis of different side chain lengths of ionic liquids.

I. IL with MSO anions

As shown in Scheme 1, 1 equivalence (eq) of reaction alcohol (methanol, propanol, pentanol, heptanol, decanol, or dodecanol) and 1.2 eq of triethylamine were added to a reaction bulb, and then 30 mL of dichloromethane (DCM) was added, and stirred in an ice bath for 5 min. Methane sulfonyl chloride (MsCl) at 1.1 eq was slowly dropped into the reaction bulb to produce a mixture. After that, the ice bath was removed, and the mixture was reacted at room temperature for 20 min to give the first reaction mixture.

The first reaction mixture was extracted with a 10% (w/v) citric acid aqueous solution three times (water phase), and then extracted with a 10% sodium hydrogen carbonate ($NaHCO_3$) aqueous solution three times to produce an extract. After extraction, the solvent in the extract was removed by concentration under reduced pressure. 1,2-Dimethyl imidazole at 0.9 eq and 50 mL of acetonitrile (ACN) were

added and heated to react at 60 °C for 12 h to produce a second reaction mixture. After that, the solvent in the second reaction mixture was removed by concentration under reduced pressure to provide the first crude product. The first crude product was extracted with hexane, and dried by concentration under reduced pressure.

Name	State	yield
[TMIM][MSO]	Yellow oil	22%
[C3DMIM][MSO]	Yellow oil	95%
[C5DMIM][MSO]	White solid	95%
[C7DMIM][MSO]	White solid	99%
[C10DMIM][MSO]	Yellow solid	98%
[C12DMIM][MSO]	Yellow solid	99%

Table S1. Summary of yield and product state for imidazolium ionic liquid with MSO anion

NMR:

[TMIM][MSO]

NMR (200 MHz, $CDCl_3$): 2.8 (3H, s), 2.91 (3H, s), 4.0 (6H, s), 7.4-7.6 (1H, d),7.8-7.9 (1H, d).

[C3DMIM][MSO]

NMR (200 MHz, $CDCl_3$): 0.88 (3H, t), 1.89-1.91 (2H, t), 2.84 (3H, s), 3.84-3.85 (2H, t), 3.91 (3H, s), 4.11 (3H, s), 7.64-7.65 (1H, d), 7.83-7.84 (1H, s).

[C5DMIM][MSO]

NMR (200 MHz, CDCl3): 086-0.88 (3H, t), 1.86-1.91 (4H, m), 2.85 (3H, s), 3.85-3.87 (2H, t), 3.91 (3H, s), 4.12 (3H, s), 7.84-7.85 (1H, d), 7.87-7.88 (1H, s).

[C7DMIM][MSO]

NMR (200 MHz, CDCl3): 0.86-0.88 (3H, t), 1.22-1.29 (10H, m), 2.87 (3H, s), 3.48-3.50 (2H, t), 4.01 (3H, s), 4.28 (3H, s), 7.85-7.87 (2H, d).

[C9DMIM][MSO]

NMR (200 MHz, CDCl3): 0.84-0.86 (3H, t), 1.20-1.28 (12H, m), 1.84-1.87 (2H, m), 2.83 (3H, s), 2.95 (3H, s), 4.02-4.03 (2H, t), 4.36 (3H, s), 7.88-7.90 (2H, d).

[C12DMIM][MSO]

NMR (200 MHz, CDCl3): 0.86-0.88 (3H, t), 1.80-1.91 (18H, m), 2.84 (3H, s), 3.84-3.85 (2H, t), 3.91 (3H, s), 4.11 (3H, s),7.83-7.84 (2H, d).

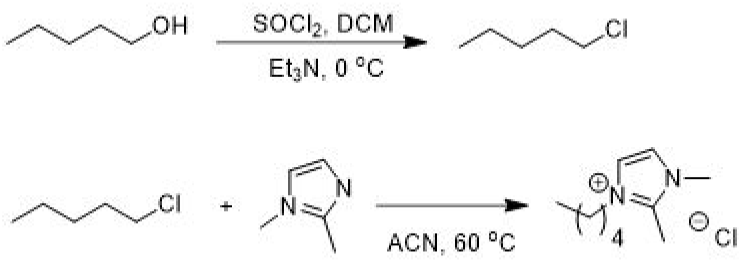

Scheme S2. Synthesis of [C5DMIM][Cl]

II. [C5DMIM][Cl]

Synthesis as show in scheme 2. One equivalence of pentanol and 30 mL of DCM were added to a bulb, and stirred in an ice bath for 5 min. Thionyl chloride ($SOCl_2$) at 1.1 eq was slowly dropped into the reaction bulb to produce a mixture. After that, the ice bath was removed, and the mixture was reacted at room temperature for 20 min to give a first reaction mixture.

Extra reactant was removed by concentration under reduced pressure. 1,2-Dimethyl imidazole at 0.9 eq and 50 mL of ACN were added and heated to react at 60 °C for 12 h to give a second reaction mixture. After that, the solvent in the second reaction mixture was removed by concentration under reduced pressure to give a first

crude product. The first crude product was extracted with hexane, and dried by concentration under reduced pressure to yield a solid yellow product [pentyl dimethyl imidazolium] [chloride] ([C5DMIm][Cl]), with a total yield of 95%.

NMR (200 MHz, $CDCl_3$): 0.87-0.89 (3H, t), 1.85-1.91 (4H, m), 3.83-3.86 (2H, t), 3.95 (3H, s), 4.13 (3H, s), 7.85-7.86 (1H, d), 7.87-7.88 (1H, s).

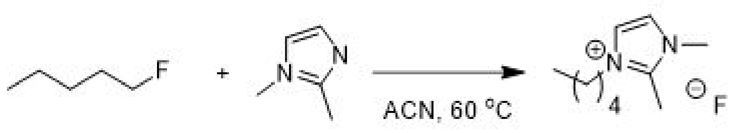

Scheme S3. Synthesis of [C5DMIM][F]

III. [C5DMIM][F]

Synthesis as show in scheme 3. One equivalence of 1-fluoro pentane and 0.9 eq of 1,2-dimethyl imidazole were added to a bulb. Then 20 ml of ACN was added to the bulb and refluxed overnight. The first crude product was extracted with hexane, and dried by concentration under reduced pressure to yield a solid yellow product [pentyl dimethyl imidazolium][fluoride], with a total yield of 70%.

NMR (200 MHz, $CDCl_3$): 0.87-0.89 (3H, t), 1.85-1.91 (4H, m), 3.83-3.86 (2H, t), 3.95 (3H, s), 4.25 (3H, s), 7.85-7.86 (1H, d), 7.87-7.88 (1H, s).

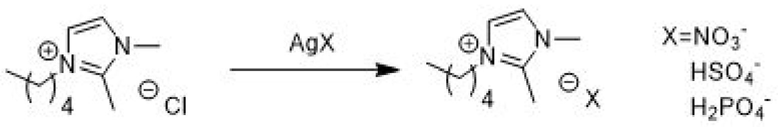

Scheme S4 Synthesis of [C5DMIM] with different anions

IV. [C5DMIM][N03]

Synthesis as show in scheme 4. One equivalence of [C5DMIM][Cl] was dissolvent in 20 ml of DI water, and 1.2 eq of silver nitrate was added and stirred for 30 min at room temperature. The precipitate was removed by 0.22-μm filtration and dried by a vacuum to yield a solid yellow product [pentyl dimethyl imidazolium][nitrate], with a total yield of 98%.

NMR (200 MHz, D_2O): 0.86-0.88 (3H, t), 1.23-1.26 (8H, m), 1,27-1.30 (2H, t), 4.01 (3H, s), 4.28 (3H, s), 7.86-7.87 (2H, d).

V. [C5DMIM][H2PO4]

One equivalence of [C5DMIM][Cl] was dissolved in 20 ml of DI water, and 1.2 eq of silver dihydrogen phosphate was added and stirred for 30 min at room temperature. The precipitate was removed by 0.22-μm filtration and dried by a vacuum to yield a solid yellow product [pentyl dimethyl imidazolium][dihydrogen phosphate], with a total yield of 98%.

NMR (200 MHz, D_2O): 0.86-0.88 (3H, t), 1.22-1.27 (8H, m), 1,28-1.31 (2H, t),

4.03 (3H, s), 4.26 (3H, s), 7.85-7.87 (2H, d).

VI. [C5DMIM][HSO4]

One equivalence of [C5DMIM][Cl] was dissolved in 20 ml of DI water, and 1.2 eq of silver hydrogen sulfonic was added and stirred for 30 min at room temperature. The precipitate was removed by 0.22-μm filtration and dried by a vacuum to yield a solid yellow product [pentyl dimethyl imidazolium][hydrogen sulfonic], with a total yield of 97%.

NMR (200 MHz, D_2O): 0.86-0.89 (3H, t), 1.22-1.26 (8H, m), 1,28-1.32 (2H, t), 4.02 (3H, s), 4.26 (3H, s), 7.85-7.87 (2H, d).

VII. [HDMIM][MSO]

One equivalence of dimethyl imidazole was dissolved in 5 ml of DI water and added to a bulb in an ice bath; 1.05 eq of methane sulfonic acid was slowly added to the bulb and reacted for 5 min. The solvent was removed by a vacuum to yield a solid white product [HDMIM][MSO], with a total yield of 99%.

NMR (200 MHz, D_2O): 2.85 (3H, s), 2.94 (3H, s), 3.75 (3H,s), 7.81 (1H, dd), 7.92 (1H, dd), 11.1 (1H, s).

	General gliadin extraction methods			In this report
	Heating extraction[a]	Solid extraction[b]	Alcohol extraction[c]	IL solution extraction
Process of extraction	>5	>5	>3	1
Time of extraction	2-4 hours	>12 hours	5 minutes	1 minute
Environmentally friendly	Yes	Yes	No	Yes
instrument	Yes (hot plate)	Yes (Shaker)	No	No
Cost	$ 1.5	$1.5	$ 1.2	$ 0.2

Table S2. Comparison of extraction methods.

a. Using heating to raise the solubility of gliadin by denature structure[3].
b. Increase solubility of gliadin by long term stirring under water solution[4].
c. Increase solubility of gliadin by alcohol solution[5].

Reference:

1. Béla Urbán,; Gábor Szalontai,; Máté Papp,; Csaba Fehér,; Attila C. Bényei,; Rita Skoda-Földes.; Characterization of the ionic liquid obtained by chlorosulfonation of 1-methylimidazole: 1-methyl-3-sulfonic acid imidazolium chloride, 1-methylimidazolium chlorosulfate or a zwitterionic salt? *Journal of molecular liquids.* **2021**, 326 (15), 115276.

2. Lenny B Malihan,; Neha Mittal,; Grace M Nisola,; Teklebrahan G Weldemhret,; Hern Kim,; Wook-Jin Chung., Macroalgal biomass hydrolysis using dicationic acidic ionic liquids. *Journal of chemical technology and biotechnology*. **2016**. 14. 1290-1297.

3. ZWEIFEL, Christoph, et al. Influence of high-temperature drying on structural and textural properties of durum wheat pasta. Cereal Chemistry, 2003, 80.2: 159-167.

4. DUPONT, Frances M., et al. Sequential extraction and quantitative recovery of gliadins, glutenins, and other proteins from small samples of wheat flour. *Journal of Agricultural and Food Chemistry*, 2005, 53.5: 1575-1584.

5. MOREL, Marie-Hélène, et al. Thermodynamic insights on the liquid-liquid fractionation of gluten proteins in aqueous ethanol. *Food Hydrocolloids*, 2022, 123: 107142.

.
